# Supplementary material for: Principled approach to the selection of the embedding dimension of networks
Source: Nat Commun. 2021 Jun 18;12:3772. doi: 10.1038/s41467-021-23795-5 (PMC8213704; doi:10.1038/s41467-021-23795-5)
Supplement: Supplementary file 1 — Supplementary Information [file 41467_2021_23795_MOESM1_ESM.pdf]

SUPPLEMENTARY INFORMATION

Principled approach to the selection of the embedding dimension of networks

Weiwei Gu, Aditya Tandon, Yong-Yeol Ahn, Filippo Radicchi

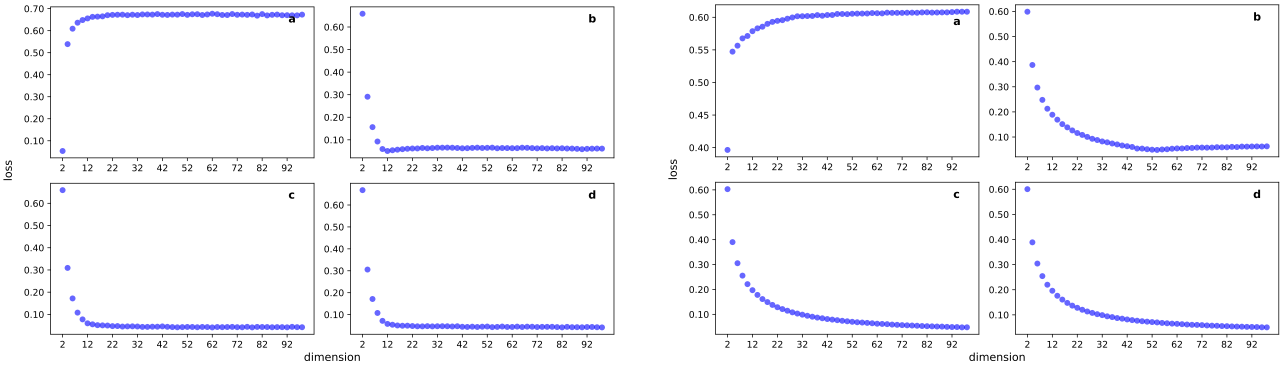

Supplementary Figure 1. Robustness of the behavior of the normalized loss function against specific choices for the value of the reference dimension. We present results obtained for different values of reference dimension d_r. In the left panels, we show results obtained for the American college football with the reference dimension 2 in panel **a**, 12 in panel **b**, 100 in panel **c** and 115 in panel **d**. The right panels show results for the Cora citation network with reference dimension of 2 in panel **a**, 52 in panel **b**, 500 in panel **c** and 2,708 in panel **d**.

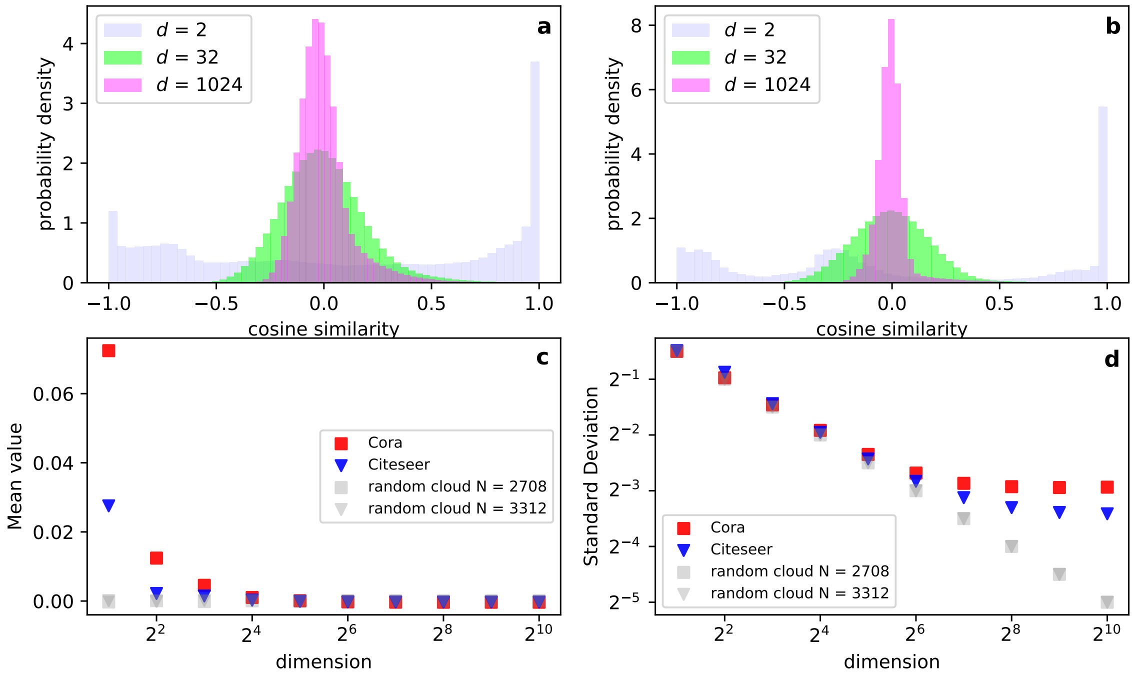

Supplementary Figure 2. Cosine similarity distribution for `node2vec` embeddings of real-world networks. **a** Distribution of the cosine similarity between pairs of nodes of the Cora citation network. We display results valid for `node2vec` embeddings in $d = 2$, $d = 32$ and $d = 1,024$ dimensions. **b** Same as in panel a, but for the Citeseer citation network. **c** Average value of the cosine similarity distribution of `node2vec` embeddings as a function of the embedding dimension. We display results for both the Cora and Citeseer citation networks. As terms of comparison, we also display result valid for clouds of points randomly scattered in d-dimensional hyper-cubes. The number of points considered to form the clouds are identical to those of the real-world networks. **d** Same as in panel c, but for the standard deviation of the cosine similarity distributions.

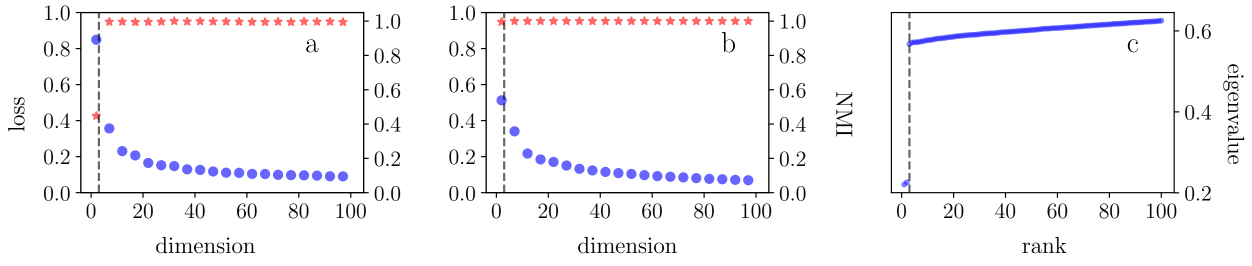

Supplementary Figure 3. Geometric embedding and community detection in synthetic graph models. We consider the SB model with $N = 3,000$ nodes and $C = 3$ communities of equal size. We set the model parameters as $p_{out} = p_{in}/10$ and $p_{in}N/C + p_{out}(C-1)N/C = 10N$. The latter equation serves to fix the expected total number of edges $M = 10N$. Results are averaged over 10 independent instances of the SB model. **a** Normalized embedding loss for `node2vec` as a function of the dimension d of the embedding space (blue circles). We plot also values of the normalized mutual information (NMI) as a function of d. NMI quantifies the performance of the k-means algorithm, based on the `node2vec` embedding of the network, to recover the ground-truth community structure (red stars). The vertical black dashed line is set at $d = C$. **b** Same as in panel a, but for the Laplacian Eigenmaps embedding. **c** We rank the eigenvalues of the graph Laplacian in ascending order, and plot their values as a function of their rank position, namely r. The black dashed line is set at $r = C$.

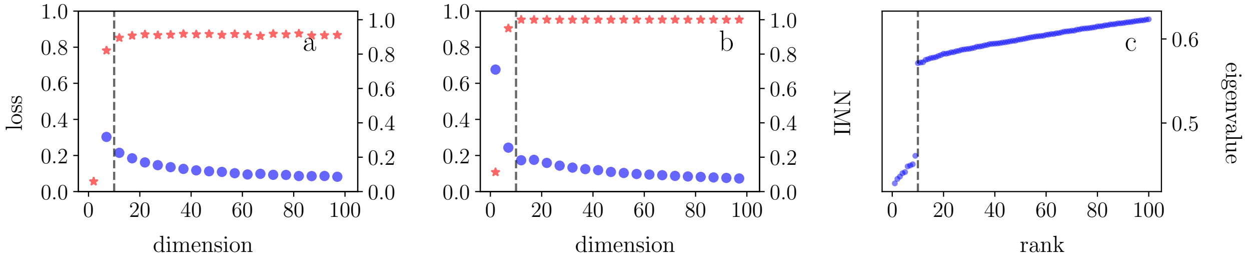

Supplementary Figure 4. Geometric embedding and community detection in synthetic graph models. Same as in Figure 3 but for the SB model with $N = 3,000$ and $C = 10$.

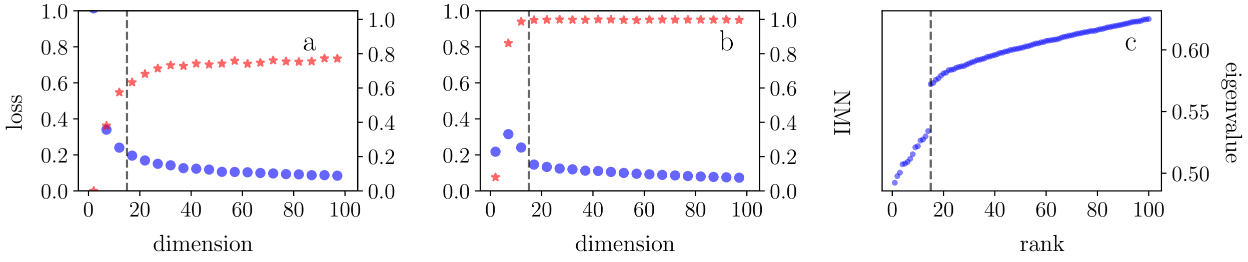

Supplementary Figure 5. Geometric embedding and community detection in synthetic graph models. Same as in Figure 3 but for the SB model with $N = 3,000$ and $C = 15$.

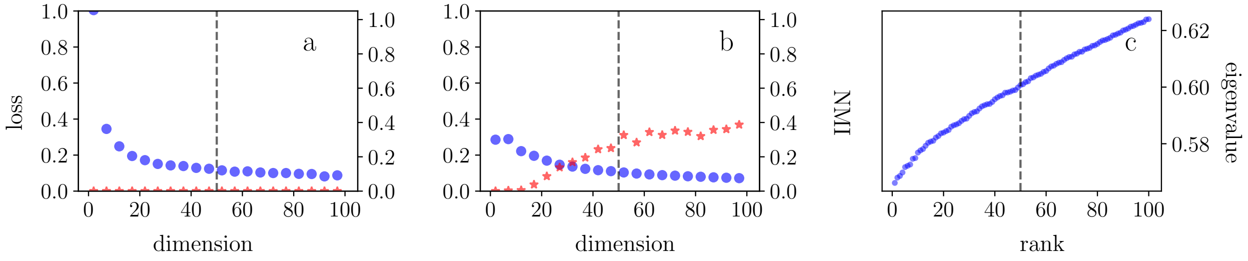

Supplementary Figure 6. Geometric embedding and community detection in synthetic graph models. Same as in Figure 3 but for the SB model with $N = 3,000$ and $C = 50$.

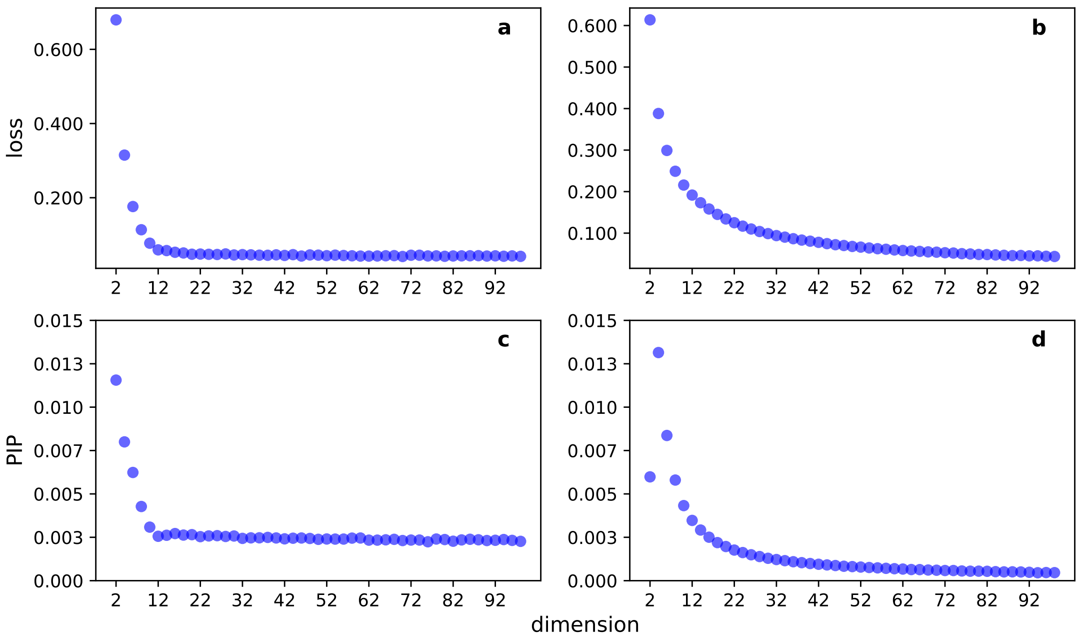

Supplementary Figure 7. Comparison between normalized loss function and PIP metric. a Normalized loss as function of the embedding dimension for the American college football network. **c** PIP loss as function of the embedding dimension for the American college football network. **b** and **d** Same as in panels a and c, respectively, but for the Cora citation network. Results are obtained using `node2vec` embeddings. Panels a and b show the same data points as of Figure(1) of the main text.

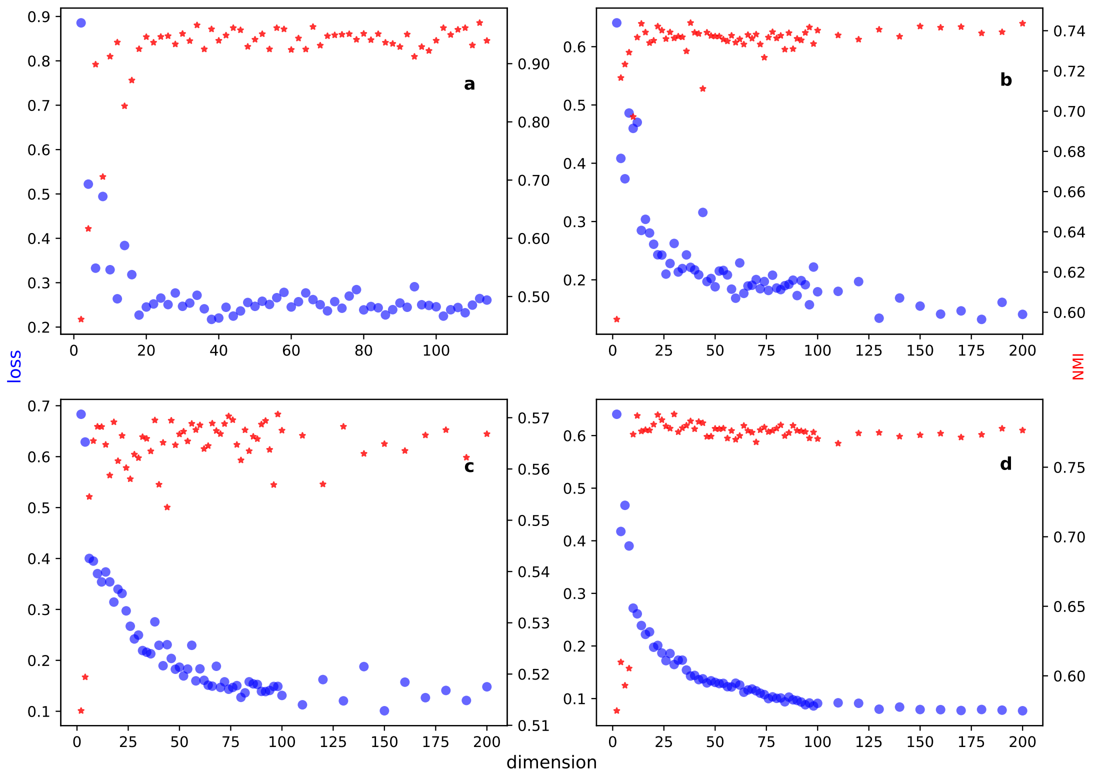

Supplementary Figure 8. `GraphSAGE` embedding and community detection in real-world networks. We consider the American college football network in panel **a**, the Citeseer citation network in panel **b**, the Cora citation network in panel **c** and the ca-GrQc network in panel **d**. Results are averaged over the 10 independent runs of the unsupervised algorithm `GraphSAGE` with mean aggregator. In our experiments, we tune the number of the units in the last layer while keeping the other parameter values unchanged. We treat the number of units in the last layer as a proxy of the embedding dimension. Blue circles represent the normalized embedding loss of `GraphSAGE` embeddings as a function of the embedding dimension. Red stars stand for the NMI value obtained by comparing the community structure identified by Infomap[1] and the ones obtained with the k-means algorithm applied to the `GraphSAGE` embeddings.

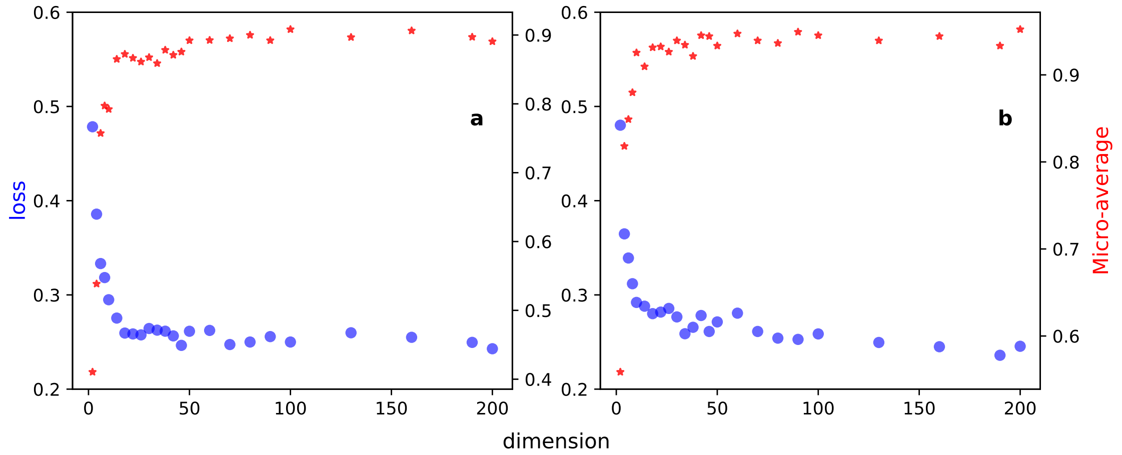

Supplementary Figure 9. `GraphRNA` embedding and node classification in real-world networks. We consider the Flickr and the BlogCatalog [2] networks in panels **a** and **b**, respectively. Nodes in the BlogCatalog network represent users and edges stand for interactions between them. Keywords of blogs serve as network attributes. Users could register their blogs into six predefined classes, which are used as the nodes' classes in our node classification task. Nodes in Flickr are users and edges indicate following relationships among them. Attributes are tags related to the photos shared by the users. The nine groups that users have joined are used as the ground-truth classification in our task. Given the learned representations from the `GraphRNA` embedding and given the labels of nodes, we leverage the training set and the corresponding labels to train a multilayer perceptron classifier. We then use the validation set to fine tune the hyperparameters of the classifier under the micro average metric. In our experiments, we treat the number of the units of the second-last layer at the same strength as the embedding dimension. All other parameters of the algorithm are kept constant. Blue circles represent the normalized embedding loss of the `GraphRNA` embeddings as a function of the embedding dimension. The micro average of the test set, displayed as red stars, quantifies the accuracy in the classification of the nodes at different values of the embedding dimension.

Supplementary Table 1. Embedding real-world networks. List of all networks analyzed in our paper. From left to right, we report: name of the network, number of nodes N, number of edges M, value of the best estimate $\hat{d}_o(\varepsilon)$ at accuracy level $\varepsilon = 0.05$, values of the best estimates $\hat{s}$, $\hat{\alpha}$ and $\hat{L}_\infty$ obtained in fitting numerical results with the function of Eq.(7) in the main paper, mean-squared error R^2 of the fit with the function of Eq.(7), reference to paper where the network data have been first considered, url to the repository where data have been downloaded. Embeddings of the networks have been performed using the algorithm `node2vec`.

network name	N	M	$\hat{d}_o(\varepsilon = 0.05)$	$\hat{s}$	$\hat{\alpha}$	$\hat{L}_\infty$	R^2	Refs.	Url
Rhesus macaques	16	69	15	1.871 ± 0.121	0.966 ± 0.160	0.000 ± 0.069	$4.3 \cdot 10^{-4}$	3	url
Highland tribes	16	58	15	1.854 ± 0.112	1.020 ± 0.139	0.000 ± 0.052	$2.9 \cdot 10^{-4}$	4	url
Kangaroos	17	91	16	1.670 ± 0.057	0.791 ± 0.101	0.000 ± 0.058	$2.3 \cdot 10^{-4}$	5	url
Crisis in a Cloister	18	126	17	1.723 ± 0.073	0.872 ± 0.108	0.000 ± 0.052	$2.6 \cdot 10^{-4}$	6	url
Southern women (large)	18	64	10	0.483 ± 0.025	0.936 ± 0.121	0.000 ± 0.014	$2.4 \cdot 10^{-5}$	7	url
Taro exchange	22	39	5	2.732 ± 0.727	2.549 ± 0.387	0.034 ± 0.009	$2.7 \cdot 10^{-4}$	8	url
Corporate leaderships	24	86	12	0.405 ± 0.038	0.506 ± 0.210	0.000 ± 0.066	$1.4 \cdot 10^{-4}$	9	url
Corporate club memberships	25	90	24	1.899 ± 0.113	1.042 ± 0.099	0.000 ± 0.028	$4.6 \cdot 10^{-4}$	10	url
Bison	26	222	25	1.719 ± 0.068	0.861 ± 0.076	0.000 ± 0.031	$3.0 \cdot 10^{-4}$	11	url
Zebra	27	111	5	0.514 ± 0.077	1.606 ± 0.220	0.008 ± 0.005	$4.1 \cdot 10^{-5}$	12	url
Cattle	28	205	27	1.690 ± 0.050	0.830 ± 0.056	0.000 ± 0.024	$1.9 \cdot 10^{-4}$	13	url
Sheep	28	235	27	1.711 ± 0.069	0.898 ± 0.071	0.000 ± 0.026	$2.9 \cdot 10^{-4}$	14	url
Seventh graders	29	250	28	1.535 ± 0.050	0.908 ± 0.055	0.000 ± 0.017	$1.6 \cdot 10^{-4}$	15	url
Hens	32	496	31	1.739 ± 0.061	0.870 ± 0.059	0.000 ± 0.022	$2.7 \cdot 10^{-4}$	16	url
Dutch college	32	422	31	1.748 ± 0.058	0.852 ± 0.057	0.000 ± 0.022	$2.6 \cdot 10^{-4}$	17	url
Zachary karate club	34	78	5	0.782 ± 0.105	1.774 ± 0.185	0.022 ± 0.004	$6.6 \cdot 10^{-5}$	18	url
Contiguous USA	49	107	5	2.801 ± 0.294	2.608 ± 0.147	0.040 ± 0.002	$6.1 \cdot 10^{-5}$	19	url
Dolphins	62	159	8	0.924 ± 0.019	1.429 ± 0.026	0.044 ± 0.001	$8.0 \cdot 10^{-6}$	20	url
Japanese macaques	62	1,167	12	1.994 ± 0.176	1.534 ± 0.111	0.216 ± 0.006	$4.9 \cdot 10^{-4}$	21	url
Train bombing	64	243	7	1.049 ± 0.068	1.649 ± 0.083	0.052 ± 0.002	$5.5 \cdot 10^{-5}$	22	url
Highschool	70	274	7	1.755 ± 0.113	1.972 ± 0.085	0.048 ± 0.002	$6.2 \cdot 10^{-5}$	23	url
Les Misérables	77	254	8	1.436 ± 0.075	1.616 ± 0.065	0.048 ± 0.002	$7.8 \cdot 10^{-5}$	24	url
David Copperfield	112	425	22	1.835 ± 0.037	1.178 ± 0.021	0.033 ± 0.002	$6.9 \cdot 10^{-5}$	25	url
Hypertext 2009	113	2,196	13	2.237 ± 0.022	1.522 ± 0.011	0.112 ± 0.001	$9.5 \cdot 10^{-6}$	26	url
the American college football	115	613	9	1.806 ± 0.036	1.468 ± 0.046	0.036 ± 0.001	$4.3 \cdot 10^{-4}$	27	url
Florida ecosystem dry	128	2,106	13	2.030 ± 0.038	1.445 ± 0.021	0.063 ± 0.001	$3.6 \cdot 10^{-5}$	28	url
Florida ecosystem wet	128	2,075	14	2.090 ± 0.039	1.440 ± 0.021	0.063 ± 0.001	$4.0 \cdot 10^{-5}$	28	url
American Revolution	136	157	4	2.136 ± 0.111	2.952 ± 0.073	0.037 ± 0.000	$3.9 \cdot 10^{-6}$	29	url
Manufacturing emails	167	3,250	17	1.814 ± 0.021	1.282 ± 0.012	0.067 ± 0.001	$1.8 \cdot 10^{-5}$	30	url
Little Rock Lake	183	2,434	11	1.836 ± 0.019	1.541 ± 0.012	0.044 ± 0.000	$7.6 \cdot 10^{-6}$	31	url
Jazz musicians	198	2,741	21	1.059 ± 0.023	1.008 ± 0.020	0.032 ± 0.001	$4.8 \cdot 10^{-5}$	32	url
PDZBase	212	242	16	1.100 ± 0.041	1.123 ± 0.037	0.026 ± 0.002	$1.1 \cdot 10^{-4}$	33	url
Residence hall	217	1,839	28	1.487 ± 0.013	1.022 ± 0.008	0.023 ± 0.001	$1.6 \cdot 10^{-5}$	34	url
Physicians	241	923	20	1.863 ± 0.033	1.217 ± 0.019	0.032 ± 0.001	$4.9 \cdot 10^{-5}$	35	url
Haggle	274	2,124	23	1.702 ± 0.022	1.131 ± 0.012	0.043 ± 0.001	$3.4 \cdot 10^{-5}$	36	url
Infectious	410	2,765	32	0.908 ± 0.014	0.838 ± 0.011	0.024 ± 0.001	$3.1 \cdot 10^{-5}$	26	url
Caenorhabditis elegans	453	2,025	43	1.144 ± 0.014	0.833 ± 0.009	0.016 ± 0.001	$3.3 \cdot 10^{-5}$	37	url
Unicode languages	614	1,248	53	1.091 ± 0.019	0.779 ± 0.016	0.000 ± 0.003	$5.9 \cdot 10^{-5}$	38	url
Crime	829	1,475	55	1.127 ± 0.021	0.763 ± 0.017	0.000 ± 0.003	$7.5 \cdot 10^{-5}$	39	url
UC Irvine forum	899	7,019	83	1.370 ± 0.012	0.742 ± 0.008	0.000 ± 0.002	$2.5 \cdot 10^{-5}$	40	url
DNC co-recipients	906	10,429	50	0.955 ± 0.014	0.755 ± 0.013	0.008 ± 0.002	$3.2 \cdot 10^{-5}$	41	url
email-Eu-core.txt	1,005	16,705	71	1.175 ± 0.007	0.743 ± 0.005	0.004 ± 0.001	$8.2 \cdot 10^{-6}$	42	url
U. Rovira i Virgili	1,133	5,450	64	1.072 ± 0.011	0.703 ± 0.009	0.000 ± 0.002	$2.3 \cdot 10^{-5}$	43	url
Euroroads	1,174	1,417	114	0.996 ± 0.010	0.608 ± 0.010	0.000 ± 0.003	$3.0 \cdot 10^{-5}$	44	url
Blogs	1,224	16,715	107	1.273 ± 0.006	0.646 ± 0.005	0.000 ± 0.001	$9.9 \cdot 10^{-6}$	45	url
Air traffic control	1,226	2,408	75	0.966 ± 0.013	0.683 ± 0.012	0.000 ± 0.003	$3.8 \cdot 10^{-5}$	46	url
Venture Capital	1,436	4,623	67	1.079 ± 0.013	0.712 ± 0.011	0.000 ± 0.002	$3.4 \cdot 10^{-5}$	47	url
Chicago	1,467	1,298	88	0.958 ± 0.018	0.636 ± 0.018	0.000 ± 0.004	$8.6 \cdot 10^{-5}$	48	url
US airports	1,574	17,215	94	1.018 ± 0.007	0.659 ± 0.006	0.000 ± 0.002	$1.2 \cdot 10^{-5}$	49	url
Human proteins (Stelzl)	1,702	3,155	65	1.054 ± 0.015	0.701 ± 0.013	0.000 ± 0.003	$4.8 \cdot 10^{-5}$	50	url
Bible	1,773	9,131	71	1.011 ± 0.011	0.659 ± 0.010	0.000 ± 0.002	$2.9 \cdot 10^{-5}$	51	url
Hamsterster friendships	1,858	12,534	73	1.053 ± 0.009	0.671 ± 0.007	0.000 ± 0.002	$1.7 \cdot 10^{-5}$	52	url
Yeast	1,870	2,277	72	1.028 ± 0.013	0.708 ± 0.012	0.003 ± 0.002	$3.5 \cdot 10^{-5}$	53	url

DNC emails	1,891	4,465	52	0.967 ± 0.009	0.752 ± 0.008	0.009 ± 0.001	$1.5 \cdot 10^{-5}$	[54]	url
UC Irvine messages	1,899	13,838	94	1.309 ± 0.013	0.718 ± 0.009	0.000 ± 0.002	$3.4 \cdot 10^{-5}$	[55]	url
Human proteins (Figeys)	2,239	6,432	73	1.026 ± 0.012	0.648 ± 0.010	0.000 ± 0.003	$3.4 \cdot 10^{-5}$	[56]	url
Hamsterster full	2,426	16,631	75	0.995 ± 0.009	0.643 ± 0.008	0.000 ± 0.002	$2.1 \cdot 10^{-5}$	[57]	url
Adolescent health	2,539	10,455	78	0.938 ± 0.008	0.548 ± 0.008	0.000 ± 0.003	$2.4 \cdot 10^{-5}$	[58]	url
Cora citation	2,708	5,429	44	1.030 ± 0.006	0.801 ± 0.009	0.048 ± 0.002	$1.1 \cdot 10^{-4}$	[42]	url
Facebook (NIPS)	2,888	2,981	10	1.317 ± 0.057	1.476 ± 0.050	0.027 ± 0.001	$7.7 \cdot 10^{-5}$	[59]	url
OpenFlights (Opsahl)	2,939	15,677	73	0.938 ± 0.007	0.684 ± 0.007	0.006 ± 0.001	$1.2 \cdot 10^{-5}$	[60]	url
Human proteins (Vidal)	3,133	6,726	72	1.028 ± 0.011	0.662 ± 0.010	0.000 ± 0.002	$2.8 \cdot 10^{-5}$	[61]	url
Citeseer citation	3,312	4,732	114	0.985 ± 0.007	0.628 ± 0.007	0.110 ± 0.002	$1.3 \cdot 10^{-5}$	[62]	url
OpenFlights (Patokallio)	3,425	19,256	68	0.950 ± 0.005	0.698 ± 0.005	0.009 ± 0.001	$5.7 \cdot 10^{-6}$	[63]	url
US power grid	4,941	6,594	145	0.970 ± 0.006	0.545 ± 0.006	0.000 ± 0.002	$1.4 \cdot 10^{-5}$	[15]	url
ca-GrQc	5,242	14,496	85	0.997 ± 0.007	0.638 ± 0.007	0.051 ± 0.002	$1.4 \cdot 10^{-5}$	[42]	url
MovieLens 1M	6,040	987,091	169	1.080 ± 0.003	0.389 ± 0.004	0.000 ± 0.003	$7.9 \cdot 10^{-6}$	[64]	url
JUNG/Javax	6,120	50,290	118	1.063 ± 0.007	0.631 ± 0.006	0.000 ± 0.002	$1.1 \cdot 10^{-5}$	[65]	url
p2p-Gnutella08.txt	6,301	20,777	108	1.057 ± 0.008	0.594 ± 0.007	0.000 ± 0.002	$1.9 \cdot 10^{-5}$	[42]	url
Reactome	6,327	147,547	67	0.967 ± 0.014	0.705 ± 0.013	0.000 ± 0.002	$4.1 \cdot 10^{-5}$	[66]	url
Java Development Kit	6,434	53,658	115	0.989 ± 0.005	0.604 ± 0.004	0.000 ± 0.001	$6.0 \cdot 10^{-6}$	[67]	url
Route views	6,474	13,895	100	1.017 ± 0.008	0.622 ± 0.007	0.000 ± 0.002	$1.6 \cdot 10^{-5}$	[68]	url
Advogato	6,539	43,277	104	1.119 ± 0.010	0.657 ± 0.008	0.000 ± 0.002	$2.3 \cdot 10^{-5}$	[69]	url
wiki-Vote.txt	7,115	100,762	128	1.030 ± 0.004	0.600 ± 0.003	0.000 ± 0.001	$4.2 \cdot 10^{-6}$	[42]	url
Wikipedia elections	7,118	100,751	128	1.028 ± 0.004	0.598 ± 0.003	0.000 ± 0.001	$4.3 \cdot 10^{-6}$	[70]	url
Chess	7,301	55,898	101	0.978 ± 0.005	0.601 ± 0.005	0.000 ± 0.001	$8.1 \cdot 10^{-6}$	[71]	url
MovieLens user–item	7,601	55,384	94	1.331 ± 0.016	0.723 ± 0.011	0.010 ± 0.003	$4.7 \cdot 10^{-5}$	[72]	url
p2p-Gnutella09.txt	8,114	26,013	114	1.063 ± 0.009	0.596 ± 0.008	0.000 ± 0.002	$2.3 \cdot 10^{-5}$	[42]	url
p2p-Gnutella06.txt	8,717	31,525	126	1.093 ± 0.011	0.610 ± 0.009	0.000 ± 0.003	$3.1 \cdot 10^{-5}$	[42]	url
p2p-Gnutella05.txt	8,846	31,839	126	1.109 ± 0.011	0.617 ± 0.009	0.000 ± 0.003	$3.5 \cdot 10^{-5}$	[42]	url
ca-HepTh.txt	9,877	25,998	95	0.977 ± 0.005	0.594 ± 0.005	0.000 ± 0.001	$7.5 \cdot 10^{-6}$	[42]	url
Sexual escorts	10,106	39,016	113	1.144 ± 0.014	0.662 ± 0.011	0.001 ± 0.003	$4.4 \cdot 10^{-5}$	[73]	url
Pretty Good Privacy	10,680	24,316	90	0.980 ± 0.009	0.662 ± 0.008	0.022 ± 0.002	$1.8 \cdot 10^{-5}$	[74]	url
p2p-Gnutella04.txt	10,876	39,994	133	1.150 ± 0.013	0.642 ± 0.011	0.002 ± 0.003	$4.4 \cdot 10^{-5}$	[42]	url
ca-HepPh.txt	12,008	118,521	90	0.687 ± 0.004	0.482 ± 0.005	0.000 ± 0.002	$6.2 \cdot 10^{-6}$	[42]	url
DBLP	12,591	49,620	95	0.989 ± 0.005	0.635 ± 0.004	0.000 ± 0.001	$5.5 \cdot 10^{-6}$	[75]	url
Google.com internal	15,763	148,585	176	0.791 ± 0.008	0.393 ± 0.012	0.000 ± 0.007	$4.4 \cdot 10^{-5}$	[76]	url
MovieLens user–tag	16,528	43,739	113	0.987 ± 0.005	0.586 ± 0.004	0.000 ± 0.001	$6.6 \cdot 10^{-6}$	[77]	url
MovieLens tag–item	16,528	71,067	125	1.137 ± 0.012	0.647 ± 0.010	0.001 ± 0.003	$3.7 \cdot 10^{-5}$	[78]	url
arXiv astro-ph	18,771	198,050	65	0.741 ± 0.004	0.598 ± 0.005	0.000 ± 0.001	$5.0 \cdot 10^{-6}$	[68]	url
ca-AstroPh.txt	18,772	198,110	66	0.759 ± 0.004	0.613 ± 0.005	0.000 ± 0.001	$5.5 \cdot 10^{-6}$	[42]	url
arXiv cond-mat	22,015	58,586	121	0.981 ± 0.006	0.562 ± 0.005	0.000 ± 0.002	$1.0 \cdot 10^{-5}$	[79]	url
p2p-Gnutella25.txt	22,687	54,705	142	1.039 ± 0.010	0.593 ± 0.009	0.000 ± 0.003	$2.7 \cdot 10^{-5}$	[42]	url
ca-cit-HepTh	22,908	2,444,798	30	0.823 ± 0.009	0.830 ± 0.010	0.005 ± 0.001	$1.2 \cdot 10^{-5}$	[42]	url
Edinburgh Associative Thesaurus	23,132	297,094	90	0.857 ± 0.003	0.565 ± 0.004	0.000 ± 0.001	$3.6 \cdot 10^{-6}$	[80]	url
ca-CondMat.txt	23,133	93,497	74	0.677 ± 0.003	0.569 ± 0.004	0.000 ± 0.001	$3.0 \cdot 10^{-6}$	[42]	url
Cora	23,166	89,157	107	0.973 ± 0.005	0.597 ± 0.005	0.000 ± 0.001	$6.6 \cdot 10^{-6}$	[81]	url
Twitter lists	23,370	32,831	100	0.991 ± 0.014	0.649 ± 0.013	0.015 ± 0.003	$4.9 \cdot 10^{-5}$	[82]	url
Google+ (NIPS)	23,628	39,194	71	0.980 ± 0.018	0.699 ± 0.016	0.001 ± 0.003	$6.4 \cdot 10^{-5}$	[83]	url
Wikinews edits (fr)	25,042	68,675	45	0.937 ± 0.021	0.774 ± 0.021	0.001 ± 0.003	$7.5 \cdot 10^{-5}$	[84]	url
CAIDA	26,475	53,381	117	0.941 ± 0.006	0.592 ± 0.006	0.000 ± 0.002	$1.0 \cdot 10^{-5}$	[68]	url
p2p-Gnutella24.txt	26,518	65,369	150	1.057 ± 0.011	0.604 ± 0.010	0.000 ± 0.003	$3.3 \cdot 10^{-5}$	[42]	url
Linux kernel mailing list replies	26,885	159,996	97	0.609 ± 0.007	0.422 ± 0.012	0.000 ± 0.005	$2.6 \cdot 10^{-5}$	[85]	url
Linux sources	30,834	213,217	69	0.785 ± 0.011	0.584 ± 0.013	0.000 ± 0.003	$3.7 \cdot 10^{-5}$	[86]	url
arXiv hep-ph	34,546	420,877	42	0.669 ± 0.007	0.694 ± 0.010	0.000 ± 0.001	$1.1 \cdot 10^{-5}$	[68]	url
Internet topology	34,761	107,720	118	0.952 ± 0.005	0.592 ± 0.004	0.000 ± 0.001	$6.2 \cdot 10^{-6}$	[87]	url
p2p-Gnutella30.txt	36,682	88,328	156	1.035 ± 0.010	0.598 ± 0.009	0.000 ± 0.003	$3.1 \cdot 10^{-5}$	[42]	url
Reuters-21578	38,677	978,158	82	1.387 ± 0.019	0.756 ± 0.012	0.024 ± 0.003	$6.0 \cdot 10^{-5}$	[88]	url
Jester 150	50,692	1,727,574	168	1.407 ± 0.009	0.556 ± 0.006	0.000 ± 0.003	$2.7 \cdot 10^{-5}$	[89]	url
Slashdot	51,083	116,573	135	1.015 ± 0.009	0.586 ± 0.008	0.000 ± 0.002	$2.2 \cdot 10^{-5}$	[90]	url
Brightkite	58,228	214,078	70	0.699 ± 0.005	0.598 ± 0.006	0.000 ± 0.001	$6.3 \cdot 10^{-6}$	[91]	url
Brightkite	58,228	214,078	71	0.690 ± 0.005	0.589 ± 0.006	0.000 ± 0.001	$6.1 \cdot 10^{-6}$	[91]	url
Gnutella (31)	62,586	147,892	166	1.020 ± 0.009	0.590 ± 0.008	0.001 ± 0.003	$2.4 \cdot 10^{-5}$	[92]	url
Facebook (WOSN)	63,731	817,035	67	0.741 ± 0.006	0.616 ± 0.008	0.000 ± 0.002	$1.0 \cdot 10^{-5}$	[93]	url
Jester 100	73,421	4,133,685	200	1.453 ± 0.033	0.545 ± 0.024	0.000 ± 0.014	$3.2 \cdot 10^{-4}$	[94]	url
Epinions	75,879	405,740	143	0.988 ± 0.008	0.597 ± 0.007	0.000 ± 0.002	$1.7 \cdot 10^{-5}$	[95]	url
Actors (DBpedia)	81,085	281,374	138	1.054 ± 0.016	0.619 ± 0.016	0.007 ± 0.005	$5.9 \cdot 10^{-5}$	[96]	url

vi.sualize.us user–tag	82,035	447,715	136	1.019 ± 0.014	0.614 ± 0.014	0.003 ± 0.004	$4.2 \cdot 10^{-5}$	[97]	url
Enron	86,978	297,456	114	0.910 ± 0.014	0.613 ± 0.016	0.021 ± 0.004	$4.7 \cdot 10^{-5}$	[98]	url
TV Tropes	87,677	3,217,698	119	1.042 ± 0.017	0.635 ± 0.017	0.014 ± 0.005	$6.2 \cdot 10^{-5}$	[99]	url
Prosper loans	89,269	3,330,022	161	1.031 ± 0.007	0.593 ± 0.007	0.000 ± 0.002	$1.1 \cdot 10^{-5}$	[94]	url
Writers	89,356	144,330	143	0.957 ± 0.007	0.595 ± 0.007	0.009 ± 0.002	$1.0 \cdot 10^{-5}$	[100]	url
Wikipedia threads (de)	90,153	727,870	104	1.199 ± 0.014	0.686 ± 0.011	0.015 ± 0.003	$4.4 \cdot 10^{-5}$	[94]	url
Wikiquote edits (en)	93,445	238,421	156	0.901 ± 0.006	0.554 ± 0.007	0.000 ± 0.002	$1.1 \cdot 10^{-5}$	[101]	url
Livemocha	104,103	2,193,083	99	1.259 ± 0.014	0.703 ± 0.010	0.019 ± 0.002	$3.9 \cdot 10^{-5}$	[102]	url
Github	120,865	439,858	218	0.725 ± 0.003	0.493 ± 0.005	0.000 ± 0.002	$5.3 \cdot 10^{-6}$	[103]	url
Occupations	127,575	250,937	124	1.001 ± 0.006	0.622 ± 0.006	0.003 ± 0.002	$1.0 \cdot 10^{-5}$	[104]	url
Douban	154,908	327,161	165	0.962 ± 0.006	0.572 ± 0.006	0.000 ± 0.002	$1.2 \cdot 10^{-5}$	[105]	url
Wikinews edits (en)	159,990	354,105	137	0.928 ± 0.004	0.521 ± 0.005	0.000 ± 0.002	$7.7 \cdot 10^{-6}$	[106]	url
BibSonomy user–tag	204,673	453,138	154	0.961 ± 0.006	0.578 ± 0.005	0.000 ± 0.002	$9.5 \cdot 10^{-6}$	[94]	url
Teams	901,132	1,366,464	156	0.965 ± 0.007	0.586 ± 0.007	0.002 ± 0.002	$1.5 \cdot 10^{-5}$	[107]	url
Pennsylvania	1,088,092	1,541,898	144	0.978 ± 0.012	0.599 ± 0.013	0.004 ± 0.004	$3.6 \cdot 10^{-5}$	[94]	url
Youtube friendships	1,134,890	2,987,624	119	0.965 ± 0.005	0.620 ± 0.006	0.010 ± 0.002	$6.3 \cdot 10^{-6}$	[108]	url
Wikipedia talk (fr)	1,409,666	2,267,114	175	0.933 ± 0.011	0.567 ± 0.012	0.001 ± 0.004	$3.2 \cdot 10^{-5}$	[94]	url
California	1,965,206	2,766,607	177	0.966 ± 0.007	0.527 ± 0.007	0.000 ± 0.003	$1.8 \cdot 10^{-5}$	[94]	url

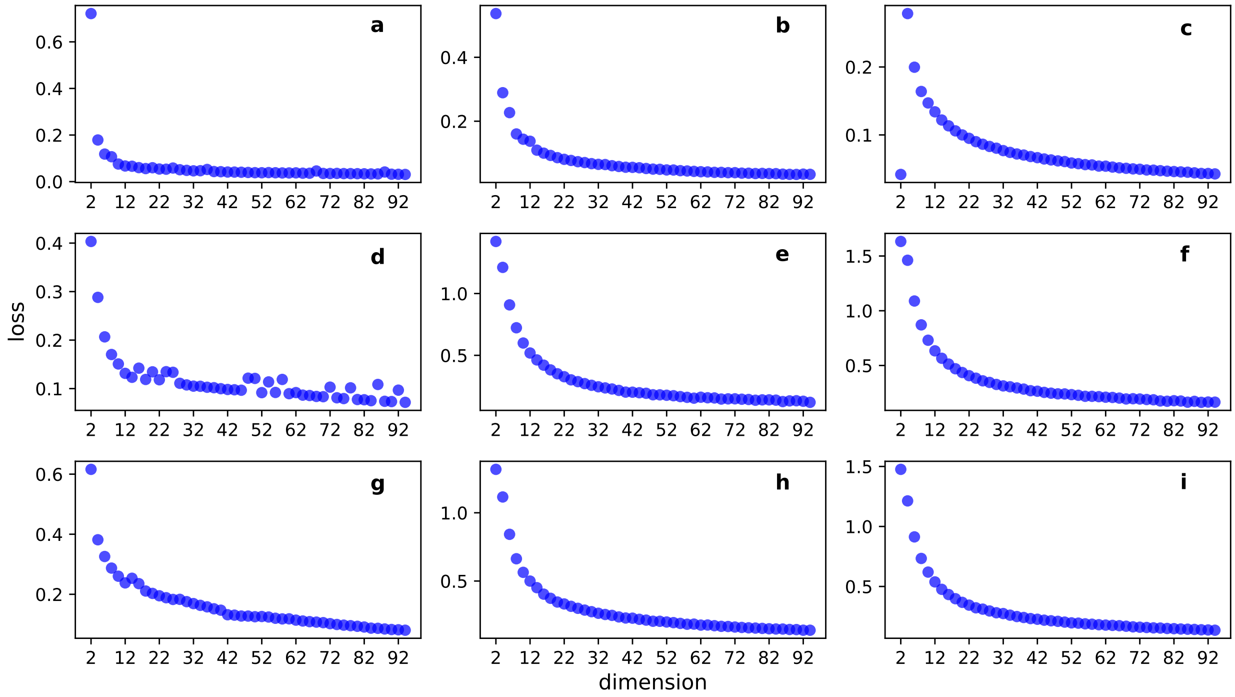

Supplementary Figure 10. Quality of network geometric embeddings depending on the definition of distance. In all panels, we plot the normalized embedding loss function for node2vec embeddings. We consider different distance metrics, and different real-world networks. The loss function of Eq.(3) of the main text is suitably modified by simply replacing cosine similarity with the distance metric under consideration. Panels **a**, **b** and **c** refer to correlation distance; **d**, **e** and **f** to Euclidean distance; panels **g**, **h** and **i** to Chebyshev distance. Panels **a**, **d** and **g** are obtained for the American college football network; panels **b**, **e** and **h** are valid the Cora citation network; panels **c**, **f** and **i** report results for the Citeseer citation network. For details on the definitions of the distances see https://docs.scipy.org/doc/scipy-0.19.1/reference/generated/scipy.spatial.distance.pdist.html.

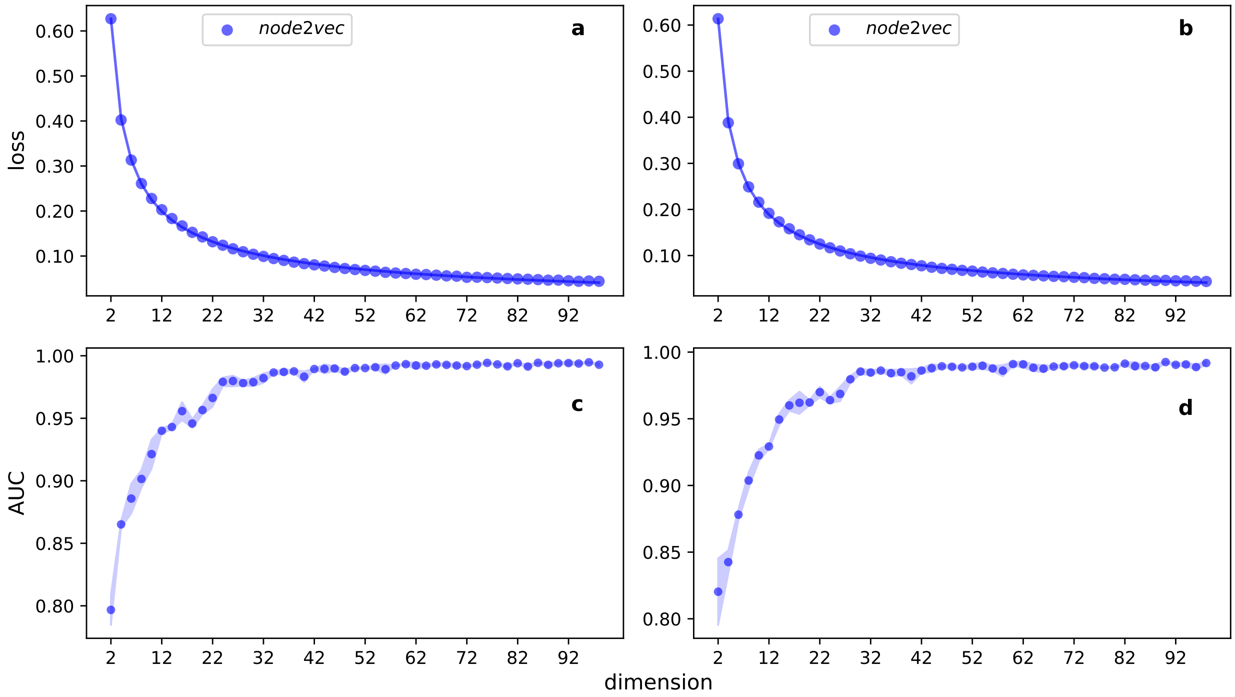

Supplementary Figure 11. Embedding of real-world networks. The description of the various panels are identical to those of Figure(1) of the main text with the difference that here we are analyzing different real-world networks. Specifically, panels **a** and **c** regard the scientific collaboration network ca-GrQc, whereas panels **b** and **d** are for the Citeseer citation network. The blue lines appearing in panels a and b are the best fits of Eq.(7) of the main paper with data points. Numerical estimates of the fitting parameters can be found in the Supplementary Table 1.

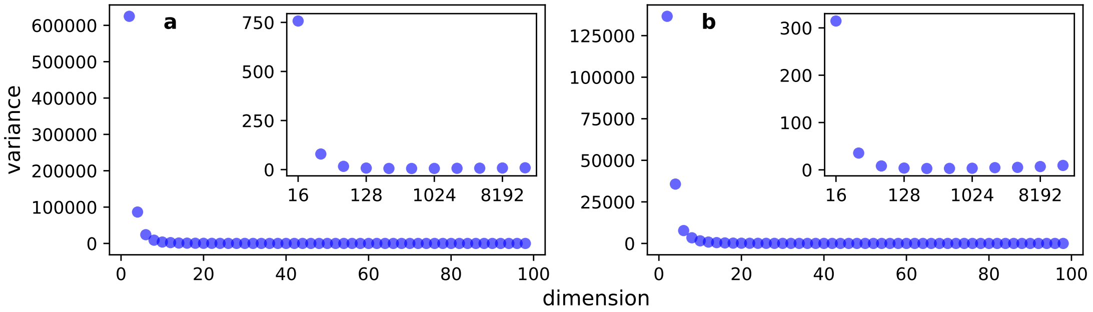

Supplementary Figure 12. Embedding variance in real networks. a Variance as a function of the embedding dimension d. Results are valid for `node2vec` embeddings of the ca-GrQc network. **b** Same as in panel a, but for the Citeseer citation network.

Supplementary References

1. Meil, M. Comparing clusterings by the variation of information. *Learn. Theory Kernel Mach.* 173–187 (2003).
2. Huang, X., Song, Q., Li, Y. & Hu, X. Graph recurrent networks with attributed random walks. In *SIGKDD Conference on Knowledge Discovery* (2019).
3. Sade, D. Sociometrics of macaca mulatta I. linkages and cliques in grooming matrices. *Folia Primatol.* **18**, 196–223 (1972).
4. Read, K. E. Cultures of the Central Highlands, New Guinea. *Southwest. J. Anthropol.* **10**, 1–43 (1954).
5. Grant, T. Dominance and association among members of a captive and a free-ranging group of grey kangaroos (*Macropus giganteus*). *Animal Behav.* **21**, 449–456 (1973).
6. Breiger, R. L., Boorman, S. A. & Arabie, P. An algorithm for clustering relational data with applications to social network analysis and comparison with multidimensional scaling. *J. Math. Psychol.* **12**, 328–383 (1975).
7. Davis, A., Gardner, B. B. & Gardner, M. R. *Deep South; a Social Anthropological Study of Caste and Class* (The University of Chicago Press, Chicago, 1941).
8. Schwimmer, E. *Exchange in the Social Structure of the Orokaiva: Traditional and Emergent Ideologies in the Northern District of Papua* (St. Martin's Press, 1973).
9. Barnes, R. & Burkett, T. Structural redundancy and multiplicity in corporate networks. *Connections* **30**, 4–20 (2010).
10. Faust, K. Centrality in affiliation networks. *Soc. Networks* **19**, 157–191 (1997).
11. Lott, D. F. Dominance relations and breeding rate in mature male American bison. *Zeitschrift für Tierpsychologie* **49**, 418–432 (1979).
12. Sundaresan, S. R., Fischhoff, I. R., Dushoff, J. & Rubenstein, D. I. Network metrics reveal differences in social organization between two fission–fusion species, Grevy's zebra and onager. *Oecologia* **151**, 140–149 (2007).
13. Schein, M. W. & Fohrman, M. H. Social dominance relationships in a herd of dairy cattle. *The Br. J. Animal Behav.* **3**, 45–55 (1955).
14. Hass, C. C. Social status in female bighorn sheep (*Ovis canadensis*): Expression, development and reproductive correlates. *J. Zool.* **225**, 509–523 (1991).
15. Watts, D. J. & Strogatz, S. H. Collective dynamics of 'small-world' networks. *Nature* **393**, 440–442 (1998).
16. Guhl, A. Social behavior of the domestic fowl. *Transactions Kansas Acad. Sci.* **71**, 379–384 (1968).
17. Van de Bunt, G. G., Van Duijn, M. A. J. & Snijders, T. A. B. Friendship networks through time: An actor-oriented dynamic statistical network model. *Comput. Math. Organ. Theory* **5**, 167–192 (1999).
18. Zachary, W. An information flow model for conflict and fission in small groups. *J. Anthropol. Res.* **33**, 452–473 (1977).
19. Knuth, D. E. *The Art of Computer Programming, Volume 4, Fascicle 0: Introduction to Combinatorial and Boolean Functions* (Addison-Wesley, 2008).
20. Lusseau, D. *et al.* The bottlenose dolphin community of Doubtful Sound features a large proportion of long-lasting associations. *Behav. Ecol. Sociobiol.* **54**, 396–405 (2003).
21. Takahata, Y. Diachronic changes in the dominance relations of adult female Japanese monkeys of the Arashiyama B group. *The Monkeys Arashiyama. State Univ. New York Press. Albany* 123–139 (1991).
22. Hayes, B. Connecting the dots. can the tools of graph theory and social-network studies unravel the next big plot? *Am. Sci.* **94**, 400–404 (2006).
23. Coleman, J. S. Introduction to mathematical sociology. *Introd. to mathematical sociology* (1964).
24. Knuth, D. E. *The Stanford GraphBase: A Platform for Combinatorial Computing*, vol. 37 (Addison-Wesley Reading, 1993).
25. Newman, M. E. J. Finding community structure in networks using the eigenvectors of matrices. *Phys. Rev. E* **74**, 036104 (2006).
26. Isella, L. *et al.* What's in a crowd? analysis of face-to-face behavioral networks. *J. Theor. Biol.* **271**, 166–180 (2011).
27. Girvan, M. & Newman, M. E. Community structure in social and biological networks. *Proc. national academy sciences* **99**, 7821–7826 (2002).
28. Ulanowicz, R. E., Heymans, J. J. & Egnotovich, M. S. Network analysis of trophic dynamics in south florida ecosystems. *US Geol. Surv. Program on South Fla. Ecosyst.* **114** (2000).
29. American revolution network dataset – KONECT (2017).
30. Manufacturing emails network dataset – KONECT (2017).
31. Martinez, N. D., Magnuson, J. J., Kratz, T. & Sierszen, M. Artifacts or attributes? effects of resolution on the Little Rock Lake food web. *Ecol. Monogr.* **61**, 367–392 (1991).
32. Gleiser, P. M. & Danon, L. Community structure in jazz. *Adv. Complex Syst.* **6**, 565–573 (2003).
33. Beuming, T., Skrabanek, L., Niv, M. Y., Mukherjee, P. & Weinstein, H. PDZBase: A protein–protein interaction database for PDZ-domains. *Bioinformatics* **21**, 827–828 (2005).
34. Freeman, L. C., Webster, C. M. & Kirke, D. M. Exploring social structure using dynamic three-dimensional color images. *Soc. Networks* **20**, 109–118 (1998).
35. Coleman, J., Katz, E. & Menzel, H. The diffusion of an innovation among physicians. *Sociometry* 253–270 (1957).
36. Chaintreau, A. *et al.* Impact of human mobility on opportunistic forwarding algorithms. *IEEE Trans. on Mob. Comput.* **6**, 606–620 (2007).
37. Duch, J. & Arenas, A. Community detection in complex networks using extremal optimization. *Phys. Rev. E* **72**, 027104 (2005).
38. Unicode languages network dataset – KONECT (2017).

39. Crime network dataset – KONECT (2017).

40. Opsahl, T. & Panzarasa, P. Triadic closure in two-mode networks: Redefining the global and local clustering coefficients. *Soc. Networks* **35**, 159–167 (2013).

41. Dnc emails co-recipients network dataset – KONECT (2017).

42. Leskovec, J. & Krevl, A. SNAP Datasets: Stanford large network dataset collection. http://snap.stanford.edu/data (2014).

43. Guimerà, R., Danon, L., Díaz-Guilera, A., Giralt, F. & Arenas, A. Self-similar community structure in a network of human interactions. *Phys. Rev. E* **68**, 065103 (2003).

44. Šubelj, L. & Bajec, M. Robust network community detection using balanced propagation. *Eur. Phys. J. B* **81**, 353–362 (2011).

45. Blogs network dataset – KONECT (2017).

46. Air traffic control network dataset – KONECT (2017).

47. Gu, W., Luo, J.-d. & Liu, J. Exploring small-world network with an elite-clique: Bringing embeddedness theory into the dynamic evolution of a venture capital network. *Soc. Networks* **57**, 70–81 (2019).

48. Boyce, D. E. *et al.* Implementation and evaluation of combined models of urban travel and location on a sketch planning network. *Chic. Area Transp. Study* 155–169 (1985).

49. Us airports network dataset – KONECT (2017).

50. Stelzl, U. *et al.* A human protein–protein interaction network: A resource for annotating the proteome. *Cell* **122**, 957–968 (2005).

51. Bible network dataset – KONECT (2017).

52. Hamsterster friendships network dataset – KONECT (2017).

53. Stumpf, M. P., Wiuf, C. & May, R. M. Subnets of scale-free networks are not scale-free: Sampling properties of networks. *Proc. Natl. Acad. Sci. United States Am.* **102**, 4221–4224 (2005).

54. Dnc emails network dataset – KONECT (2017).

55. Opsahl, T. & Panzarasa, P. Clustering in weighted networks. *Soc. Networks* **31**, 155–163 (2009).

56. Ewing, R. M. *et al.* Large-scale mapping of human protein–protein interactions by mass spectrometry. *Mol. Syst. Biol.* **3**, 89 (2007).

57. Hamsterster full network dataset – KONECT (2017).

58. Moody, J. Peer influence groups: Identifying dense clusters in large networks. *Soc. Networks* **23**, 261–283 (2001).

59. Facebook (nips) network dataset – KONECT (2017).

60. Opsahl, T., Agneessens, F. & Skvoretz, J. Node centrality in weighted networks: Generalizing degree and shortest paths. *Soc. Networks* **3**, 245–251 (2010).

61. Rual, J.-F. *et al.* Towards a proteome-scale map of the human protein–protein interaction network. *Nature* 1173–1178 (2005).

62. Giles, C. L., Bollacker, K. D. & Lawrence, S. Citeseer: An automatic citation indexing system. In *ACM DL*, 89–98 (ACM Press, 1998).

63. Openflights network dataset – KONECT (2017).

64. Movielens 1m network dataset – KONECT (2017).

65. Jung and javax dependency network dataset – KONECT (2017).

66. Joshi-Tope, G. *et al.* Reactome: A knowledgebase of biological pathways. *Nucleic Acids Res.* **33**, D428–D432 (2005).

67. Jdk dependency network dataset – KONECT (2017).

68. Leskovec, J., Kleinberg, J. & Faloutsos, C. Graph evolution: Densification and shrinking diameters. *ACM Trans. Knowl. Discov. from Data* **1**, 1–40 (2007).

69. Advogato network dataset – KONECT (2017).

70. Wikipedia elections network dataset – KONECT (2017).

71. Chess network dataset – KONECT (2017).

72. Movielens user–movie network dataset – KONECT (2017).

73. Rocha, L. E. C., Liljeros, F. & Holme, P. Information dynamics shape the sexual networks of Internet-mediated prostitution. *Proc. Natl. Acad. Sci.* **107**, 5706–5711 (2010).

74. Boguñá, M., Pastor-Satorras, R., Díaz-Guilera, A. & Arenas, A. Models of social networks based on social distance attachment. *Phys. Rev. E* **70**, 056122 (2004).

75. Dblp network dataset – KONECT (2017).

76. Palla, G., Farkas, I. J., Pollner, P., Derényi, I. & Vicsek, T. Directed network modules. *New J. Phys.* **9**, 186 (2007).

77. Movielens user–tag network dataset – KONECT (2017).

78. Movielens tag–movie network dataset – KONECT (2017).

79. Newman, M. E. J. The structure of scientific collaboration networks. *Proc. Natl. Acad. Sci.* **98**, 404–409 (2001).

80. Kiss, G. R., Armstrong, C. & Milroy, R. An associative thesaurus of English and its computer analysis. *The Comput. Lit. Stud.* 153–165 (1973).

81. Cora citation network dataset – KONECT (2017).

82. Twitter lists network dataset – KONECT (2017).

83. Google+ network dataset – KONECT (2017).

84. Wikinews (fr) network dataset – KONECT (2017).

85. Linux kernel mailing list replies network dataset – KONECT (2017).

86. Linux network dataset – KONECT (2017).

87. Zhang, B., Liu, R., Massey, D. & Zhang, L. Collecting the Internet AS-level topology. *SIGCOMM Comput. Commun. Rev.* **35**, 53–61 (2005).

88. Lewis, D. D., Yang, Y., Rose, T. G. & Li, F. RCV1: A new benchmark collection for text categorization research. *J. Mach. Learn. Res.* **5**, 361–397 (2004).

89. Goldberg, K., Roeder, T., Gupta, D. & Perkins, C. Eigentaste: A constant time collaborative filtering algorithm. *Inf. Retr.* **4**, 133–151 (2001).

90. Slashdot threads network dataset – KONECT (2017).

91. Brightkite network dataset – KONECT (2017).

92. Ripeanu, M., Foster, I. & Iamnitchi, A. Mapping the Gnutella network: Properties of large-scale peer-to-peer systems and implications for system design. *IEEE Internet Comput. J.* **6** (2002).

93. Facebook friendships network dataset – KONECT (2017).

94. Kunegis, J. Konect: The koblenz network collection. WWW '13 Companion, 1343–1350 (Association for Computing Machinery, New York, NY, USA, 2013).

95. Epinions network dataset – KONECT (2017).

96. Movies network dataset – KONECT (2017).

97. vi.sualize.us user–tag network dataset – KONECT (2017).

98. Enron network dataset – KONECT (2017).

99. Tv tropes network dataset – KONECT (2017).

100. Writers network dataset – KONECT (2017).

101. Wikiquote (en) network dataset – KONECT (2017).

102. Livemocha network dataset – KONECT (2017).

103. Github network dataset – KONECT (2017).

104. Occupation network dataset – KONECT (2017).

105. Douban network dataset – KONECT (2017).

106. Wikinews (en) network dataset – KONECT (2017).

107. Teams network dataset – KONECT (2017).

108. Youtube friendship network dataset – KONECT (2017).
